# Factors associated with unsuccessful treatment outcomes among drug-resistant tuberculosis patients in Loreto, Peru, 2015–2023: A retrospective cohort study

**DOI:** 10.1371/journal.pgph.0007229

**Published:** 2026-09-09

**Authors:** Evelyn Genthell Cordova-Pisco, Tery Vasquez-Hassinger, Estela Huamán-Angeles, Karine Zevallos, George Obregon

**Affiliations:** 1 Laboratorio de Referencia Regional de Loreto, Gerencia Regional de Salud Loreto, Iquitos, Loreto, Perú; 2 Unidad de Investigación, Hospital Regional de Loreto, Iquitos, Loreto, Perú; 3 Universidad Científica del Sur, Lima, Perú; 4 Centro Nacional de Salud Pública, Instituto Nacional de Salud, Lima, Perú; 5 Facultad de Medicina Humana, Universidad Nacional de la Amazonía Peruana, Iquitos, Loreto, Perú; 6 Unidad de Postgrado y Especialización, Universidad Peruana Cayetano Heredia, Lima, Perú; Universidad Peruana Cayetano Heredia, PERU

## Abstract

In Loreto, Peru, unsuccessful treatment outcomes among patients with drug-resistant tuberculosis (DR-TB) remain high, underscoring the need to identify associated factors in this Amazonian region. This retrospective cohort study included 417 DR-TB cases registered in the national TB system in Loreto between 2015 and 2023. Loss to follow-up, death, and treatment failure were combined into a single composite outcome variable defined as unsuccessful treatment outcome. Bivariate and multivariable logistic regression analyses were performed to estimate associations between sociodemographic, clinical, and programmatic factors and this outcome. Among the 417 cases analyzed, 49.5% had unsuccessful treatment outcomes: 34.1% due to loss to follow-up, 3.6% due to treatment failure, and 11.8% due to death. Loss to follow-up was the main driver of unsuccessful outcomes, suggesting important gaps in continuity of care and adherence support within the regional health system. In bivariate analyses, unsuccessful outcomes were associated with age 18–30 years (OR: 3.7; 95% CI: 1.6–8.5; p = 0.002), TB-HIV coinfection (OR: 2.4; 95% CI: 1.2–4.6; p = 0.012), previous treatment after loss to follow-up (OR: 2.9; 95% CI: 1.6–5.2; p < 0.001), high bacillary load (+++) (OR: 1.9; 95% CI: 1.0–3.5; p = 0.041), alcoholism (OR: 4.6; 95% CI: 1.5–14.1; p = 0.006), and drug addiction (OR: 7.0; 95% CI: 2.0–24.2; p = 0.002). In the multivariable analysis, alcoholism remained independently associated with unsuccessful outcomes (aOR: 4.0; 95% CI: 1.1–14.2; p = 0.029), while individualized treatment was associated with lower odds of unsuccessful outcomes (aOR: 0.4; 95% CI: 0.1–0.9; p = 0.023). Unsuccessful treatment outcomes among DR-TB patients in Loreto were mainly driven by loss to follow-up, highlighting persistent gaps in treatment continuity and adherence support. Programmatic priorities should include decentralized access to drug-susceptibility testing, community-based adherence strategies, and integrated screening and management of alcohol use within DR-TB care.

## Introduction

Drug-resistant tuberculosis (DR-TB) is a major global public health problem. According to the 2025 Global Tuberculosis Report of the World Health Organization (WHO), a total of 158,000 cases of DR-TB were reported [1], representing a worrisome threat to achieving the TB control targets set for the end of this decade. The WHO End TB Strategy aims for an 80% reduction in incidence and a 90% reduction in TB mortality by 2030, compared with 2015 levels [1]. However, global progress toward these goals is hindered by the growing burden of DR-TB, particularly in high-burden countries such as Peru.

At a national level, between 2015 and 2023, Peru reported 272,471 cases of TB, with a mortality rate of 5.5 per 100,000 population in 2023. Cases of DR-TB have shown an upward trend, particularly during the post-COVID-19 period, with treatment success rates of 78.8% for DR-TB in 2022 [2]. The Amazon region of Loreto represents a particular concern: treatment success rates for DR-TB were only 55.7% in 2015 and 53.8% in 2022 [2], both lower than the national average. This situation highlights the substantial number of patients who are not adequately diagnosed or successfully treated, thereby perpetuating TB transmission within households and communities. These persistently low success rates suggest that progress toward the 2030 End TB targets in Loreto remains insufficient and that tailored Amazonian-specific interventions are needed to address diagnostic, adherence, and health-system barriers.

Loreto accounts for 3.4% of the national DR-TB burden and is characterized by unique sociodemographic and geographic conditions: 43.5% of its population lives in extreme poverty [3], with geographic barriers to accessing health services [4]. In this region, risk factors contributing to unsuccessful treatment outcomes, defined as loss to follow-up, death, or treatment failure, have been scarcely evaluated, yet they are critical to achieving cure, preventing transmission, and curbing the rise of resistance to anti-TB drugs.

Several studies have identified factors associated with unfavorable treatment outcomes, including age, sex, TB-Human Immunodeficiency Virus (HIV) coinfection, TB-diabetes mellitus (DM) comorbidity, high bacillary load, and alcohol and/or drug use [5–7]. These factors reflect important clinical and comorbidity-related barriers that may compromise treatment adherence and reduce the likelihood of treatment success. In Loreto, these barriers are particularly relevant because mental health services, including care for alcohol- and drug-use disorders, are poorly integrated into the TB program, leaving many patients with unaddressed comorbidities that may jeopardize adherence.

Structural and health-system barriers also play a central role in DR-TB care in this Amazonian setting. Throughout the study period, the region faced significant diagnostic challenges due to severe limitations in access to drug-susceptibility testing. The complete lack of on-site rapid molecular testing until mid-2018 forced healthcare providers to transport specimens to Lima, leading to significant delays in optimizing treatment for a population already facing logistical hurdles from difficult river transport geography. GeneXpert MTB/RIF was gradually decentralized from 2019 onward but was initially restricted to priority groups, and coverage remained incomplete in remote districts. These diagnostic limitations occur within a broader context of riverine geography, dispersed and highly mobile populations, limited access to rapid drug-susceptibility testing and second-line drugs, and insufficient integration with mental health services. Therefore, it remains unclear which patient- and program-level factors are most strongly associated with loss to follow-up, treatment failure, or death among DR-TB patients in Loreto [4,8,9].

Outside the Amazon, a Peruvian case-control study in Callao found that cavitary lesions, fibrotic tracts, and resistance to ≥5 drugs were strongly associated with MDR-TB treatment failure, while a recent global meta-analysis of isoniazid-resistant TB (Hr-TB) identified previous TB treatment and initial smear positivity as key factors associated with poor outcomes [10]. These studies are important because they confirm the role of disease severity, resistance burden, and treatment history in DR-TB prognosis. However, they were conducted in settings that differ substantially from Loreto, where unsuccessful outcomes may also be shaped by Amazonian-specific barriers. In Loreto, dispersed riverine populations, long travel times to health facilities, dependence on river transport, limited access to diagnostic services, sociocultural barriers, traditional health practices, and insufficiently adapted health services may affect timely diagnosis, treatment initiation, adherence, and follow-up. Therefore, evidence from coastal urban or non-Amazonian settings cannot be directly extrapolated to Loreto without considering this unique sociocultural and health-system context. This reinforces the need to identify locally relevant patient- and program-level factors associated with loss to follow-up, treatment failure, or death among DR-TB patients in the Peruvian Amazon [4,8,11].

The primary objective of this study was to identify factors independently associated with unsuccessful treatment outcomes among DR-TB patients in Loreto during 2015–2023. This large Amazonian cohort provides the first detailed analysis of patient and program-level factors associated with unsuccessful DR-TB treatment in Loreto, with findings intended to guide more effective programmatic interventions tailored to this context.

## Methods

### Ethical Statement

This study was conducted using secondary, anonymized data and adhered to international standards for research ethics and integrity. The study protocol was reviewed and approved by the Institutional Research Ethics Committee of the Regional Hospital of Loreto (CIEI-HRL, Approval No. ID-058-CIEI-2025). In addition, authorization to access and use the SIGTB database was obtained from GERESA Loreto, documented under Official Letter No.3679-2025-GRSL-GERESA-L/30.10.01 in response to Reference Request No.01-2025-EGCP. Both ethics bodies function as equivalent committees consistent with international ethical oversight requirements.

Given the retrospective design and exclusive use of secondary anonymized data, obtaining individual patient consent was not feasible. The Institutional Research Ethics Committee determined that informed consent was not required, in accordance with Peruvian regulations and consistent with the principles of the Declaration of Helsinki. The Institutional Research Ethics Committee formally waived the requirement for informed consent due to the use of fully anonymized secondary data. No personal identifiable information was accessed, recorded, or reported. All data were fully anonymized prior to analysis, with identifiers removed at the source database level by the regional TB strategy coordinator. Therefore, no member of the research team had access to the master key, identifiable patient lists, or personal patient data. No direct contact with patients occurred at any stage of the research. Patient confidentiality was maintained following the International Committee of Medical Journal Editors (ICMJE) guidelines on privacy and data protection. Identifying information (names, photos, or personal identifiers) was not included in this manuscript. The study adhered to the ethical principles outlined in the Belmont Report and the Declaration of Helsinki and fully complied with PLOS Global Public Health’s policies on human subjects’ research, ensuring that participant privacy, data protection, and research integrity were upheld. Additionally, this manuscript was registered on the Health Research Projects Platform (PRISA) of the National Institute of Health (INS).

### Study design

This was a retrospective cohort study of patients with a confirmed diagnosis of DR-TB, using secondary surveillance data obtained from the Tuberculosis Management Information System (Spanish acronym, SIGTB) of the Regional Health Strategy for Tuberculosis Prevention and Control in Loreto (Spanish acronym, ESR PCTB). SIGTB is a mandatory reporting platform that covers all levels of care and allows follow-up of TB cases from treatment initiation to final outcome. Data entered into the system are reviewed and validated by the regional TB strategy coordinator before final closure [9].

### Study Setting

This study was conducted in the Loreto region, located in the northeastern Peruvian Amazon. With an area of 368,852 km², approximately 28% of the national territory, Loreto is the largest region in Peru and is divided into eight provinces, of which Maynas has the highest population density, with about 559,603 inhabitants. Iquitos, the regional capital, is the most densely populated metropolis in the Amazon rainforest and the sixth largest city in Peru. The city lies along the Amazon, Nanay, and Itaya rivers and, like many surrounding riverine communities, is geographically isolated, with limited transport infrastructure and no road connection to the rest of the country. Access to Iquitos and many provincial capitals is only possible by air (small commercial flights) or river transport, which substantially constrains timely access to health services, especially in rural and remote areas [12].

The health system in Loreto consists of a network of primary-care facilities (health centers and posts), general and regional hospitals, and the Regional Reference Laboratory of Loreto (LRRL, acronym in Spanish), all coordinated by the Regional Health Directorate (GERESA, acronym in Spanish) of Loreto. Within GERESA, the Regional Health Strategy for the Prevention and Control of TB (ESR-PCTB, acronym in Spanish) works in close collaboration with the LRRL to ensure diagnosis, treatment initiation, and follow-up of patients with TB. During the study period, DR-TB patients were managed according to the national TB technical standards in effect at the time of diagnosis and treatment. These standards included standardized, empirical, or individualized regimens based on drug-susceptibility testing (DST) results and national clinical criteria; later policy updates progressively incorporated shorter all-oral regimens and newer drugs, such as bedaquiline, for eligible MDR-TB patients [13].

Throughout 2015–2023, access to rapid molecular and conventional DST in Loreto evolved gradually. Before mid-2018, the region had no on-site rapid molecular testing, and most specimens had to be shipped to Lima for culture and DST. In Amazonian rural settings, specimen referral and result return may take several weeks and, in some remote areas, more than one month, delaying diagnosis and treatment optimization. The first GeneXpert MTB/RIF platform was installed at the LRRL in 2018 and was progressively decentralized to selected facilities from 2019 onward, although coverage remained incomplete in remote districts. In addition, the COVID-19 pandemic (2020–2021) led to substantial disruptions in TB services, including temporary reductions in clinic hours, reallocation of health personnel and diagnostic resources to COVID-19 activities, and interruptions in routine follow-up, all of which may have affected access to diagnosis, continuity of care, and treatment outcomes for DR-TB patients in Loreto [14].

### Population and sample

**Fig 1:**
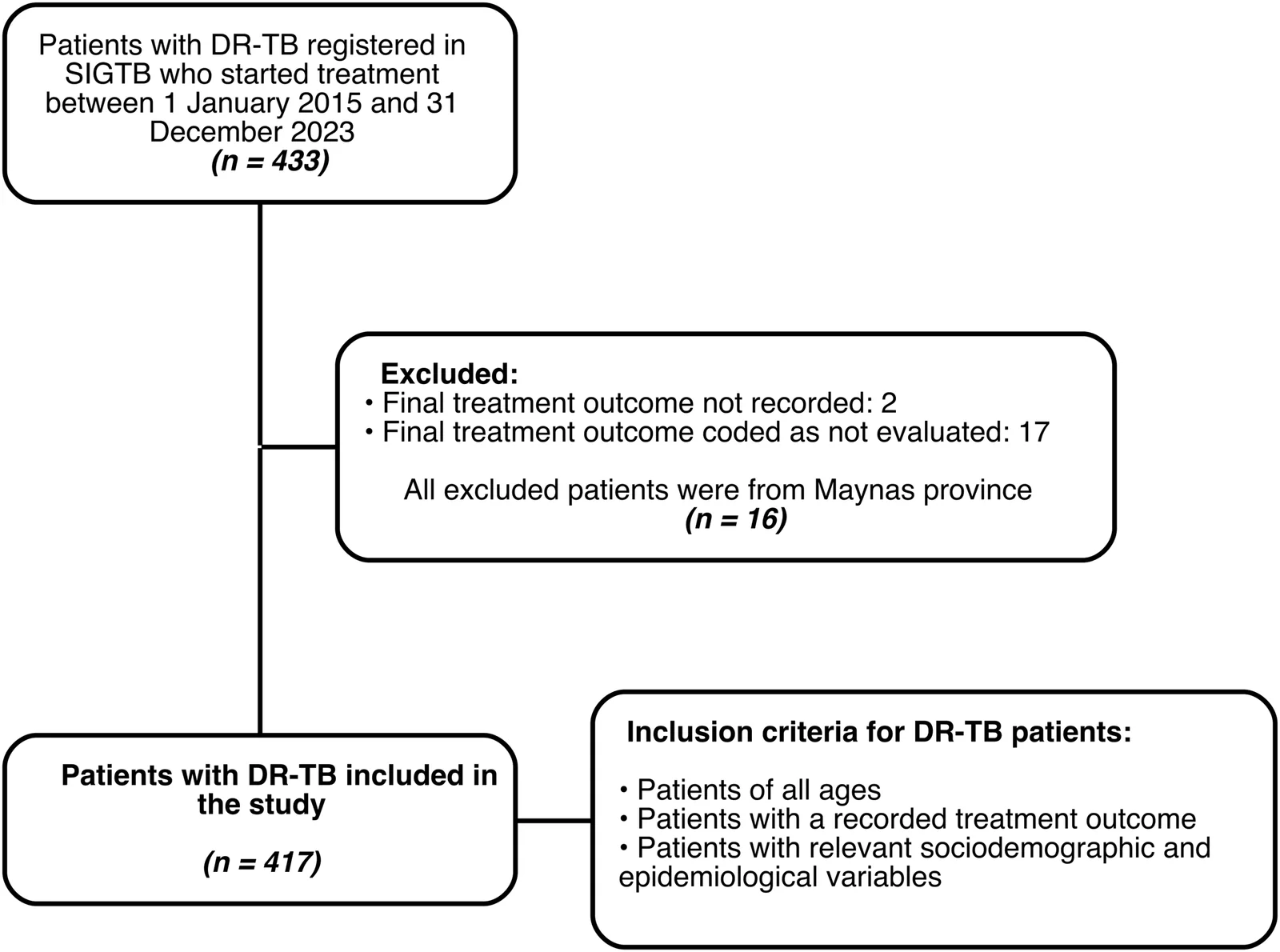
Selection of drug-resistant tuberculosis patients included in the study.

The study population included all DR-TB patients in the Loreto region who initiated treatment and were registered in the SIGTB between 1 January 2015 and 31 December 2023 (It should be noted that for the 2023 cohort, patients had already completed their treatment outcome cycle by the time the database closed, as they had a discharge status record). Patients of all ages, including pediatric cases, were eligible. A total of 433 DR-TB patients met these eligibility criteria, of whom 16 patients (3.7%) were excluded because their final treatment outcome was not recorded (two patients) or was coded as “not evaluated” (fourteen patients) in SIGTB. All excluded patients were from Maynas province; therefore, the exclusions did not disproportionately affect patients from remote provinces. This resulted in a final sample of 417 patients with documented treatment outcomes and relevant sociodemographic and epidemiological information (Fig 1).

DR-TB was defined according to the 2013 National TB Technical Standard NT N°104 as TB caused by *Mycobacterium tuberculosis* strains resistant to at least one first or second-line drug, including Hr-TB, rifampicin-resistant TB (TB-R), MDR-TB (resistance to at least isoniazid and rifampicin) and other resistance profiles as classified by the National TB Program. Drug resistance was laboratory confirmed using rapid tests for MDR-TB detection such as microscopic observation drug susceptibility (MODS), nitrate reductase assay (NRA), MGIT, and DNA probe-based assays (Genotype line-probe assays and GeneXpert MTB/RIF) and/or by conventional phenotypic drug-susceptibility testing to first and second-line drugs using the proportion method on Middlebrook 7H10 agar. The study period covered different diagnostic eras; therefore, the resistance-pattern variable should be interpreted as a composite measure influenced by changes in diagnostic availability over time, particularly the introduction of GeneXpert MTB/RIF in Loreto in 2018 and its gradual decentralization from 2019 onward.

It is important to note that the majority of registered cases came from the province of Maynas, where most of the regional population resides (85.1% of the patients included). Therefore, cases from the more remote riverine provinces may be underrepresented and lack access to diagnostic and DST due to difficult geographical access, distances, and prolonged sample transport times to the LRRL located in the city of Iquitos. This could introduce bias in the detection and regional distribution of cases.

No formal sample size calculation was performed, as this study corresponds to a census of all eligible, program-registered DR-TB cases with available outcome data in Loreto during 2015–2023.

### Procedure and data collection

Data were extracted from the SIGTB database, accessed on 25 July 2025 with authorization from the GERESA Loreto. The database did not contain personally identifiable information, ensuring patient confidentiality and compliance with institutional ethical standards. Data were organized into a collection form using Microsoft Excel® (v18.0). After extraction, the database underwent cleaning, standardization, and consistency checks by the study team before statistical analysis to minimize inconsistencies. Only variables relevant to the analysis were included and coded prior to importation into statistical software for analysis. Potential sources of bias included selection bias from excluding incomplete or inconsistent records and information bias due to reliance on secondary database entries. However, the use of a standardized national registry such as SIGTB likely reduced inter-center variability and improved comparability across reporting units. The controlled access process, coupled with ethical approval and data anonymization, ensured the robustness and transparency of the data collection process.

### Variables

The dependent variable was treatment outcome, classified according to WHO criteria as successful (cure or treatment completed) or unsuccessful (loss to follow-up, failure, or death) [15]. For the analysis, this was coded as a binary outcome (successful vs unsuccessful). The independent variables included: age, categorized as <18, 18–30, 31–45, 46–60, and >60 years; sex coded as a dichotomous variable (female/male); TB–HIV coinfection and TB–DM comorbidity, each coded as yes/no; TB treatment category at enrollment, defined according to the 2013 National TB Technical Standard NT N°104 and classified as: new case (patient who had never received TB treatment), relapse (patient previously treated and declared cured or treatment completed who developed a new episode of TB), treatment after loss to follow-up (patient with a previous episode who interrupted TB treatment and was restarted from the first dose), and failure (patient who failed a first- or second-line regimen and initiated a new therapeutic scheme); delay in treatment initiation, categorized as <14 days vs ≥ 14 days, according to National TB Program targets for timely treatment initiation after diagnosis; TB disease site, classified as pulmonary or extrapulmonary; bacillary load, categorized using the standard semi-quantitative smear scale (negative, paucibacillary, + , ++, +++); alcohol or drug use, coded as yes/no; drug-resistance pattern, categorized as Hr-TB, Rr-TB, PZAr-TB, fluoroquinolone-resistant TB, MDR-TB and others; district of residence, grouped as Maynas province (Iquitos, the main urban center) versus the remaining provinces of Loreto (peripheral/riverine districts) and treatment scheme categorized as standardized, empirical and individualized.

Operational definitions of the variables followed National TB Technical Standard NT N°104 of the Ministry of Health of Peru and WHO guidelines in effect during the study period [16]. The selected categories reflect internationally recognized epidemiological and clinical risk factors for TB outcomes and align with National TB Program reporting practices.

### Statistical analysis

Statistical analyses were performed using Stata SE version 19 (StataCorp, College Station, TX, USA). Patient characteristics were summarized using absolute and relative frequencies for categorical variables. To explore the association between independent variables and unsuccessful treatment outcome, we first performed bivariate logistic regression models to estimate crude odds ratios (ORs) with 95% confidence intervals (CIs). A p-value < 0.05 was considered statistically significant. In the multivariable logistic regression model, all variables with p ≤ 0.1 from the bivariate analysis were analyzed simultaneously. In addition, age, sex, TB–HIV coinfection, previous TB treatment history, and MDR-TB status were included in the multivariable models as potential confounders, regardless of their bivariable p-values. The interaction between TB-HIV coinfection and bacillary load was included to account for the known effect of HIV-related immunosuppression on smear positivity and to explore whether the association between bacillary load and unsuccessful outcomes differed by HIV status. Interaction terms were reported with their corresponding adjusted odds ratios and p-values. Multicollinearity was assessed using variance inflation factors, and no relevant collinearity was detected (all VIFs < 2). Model calibration and overall fit were assessed using the Hosmer-Lemeshow goodness-of-fit test and by reporting pseudo-R² and the Akaike Information Criterion. In addition, model discrimination was evaluated using the area under the receiver operating characteristic curve (AUC/c-statistic) to assess the predictive performance of the final multivariable logistic regression model. Finally, a sub-analysis was performed comparing treatment dropout to treatment success using the same variables as the main model. Patients who died or experienced treatment failure were excluded from this sub-analysis, so the comparison was restricted to loss to follow-up versus treatment success.

## Results

### Characteristics of the study population

During the study period, 417 patients were included with confirmed diagnosis of DR-TB from the Loreto region were registered in the SIGTB. The mean age of the cohort was 41.7 years (IQR: Q3 (55)-Q1 (27) =28 years), with the 31–45 years age group representing the largest proportion (27.1%). Male patients predominated in the sample (63.1%). Coinfection with HIV and DM was absent in 84.4% and 76.5% of cases, respectively. Regarding treatment history, 54.4% of patients entered the National TB Program as new cases, followed by 22.3% as treatment failures, 16.1% as treatment after loss to follow-up and 7.2% as relapses. A total of 86.3% of patients-initiated treatment within 14 days of diagnosis. Pulmonary TB was the most frequent presentation (97.1%). Negative smear microscopy at baseline was observed in 32.4% of cases. Approximately 5% of patients reported alcoholism or drug addiction. The most prevalent resistance pattern was MDR-TB (51.8%). Most patients (85.1%) came from the district of Maynas city and the most frequent treatment regimen was the empirical regimen with 66.2%. An important point is to highlight the missing data/not evaluated for some patients, as this deficient documentation suggests either patient non-compliance or a failure in the healthcare system, which would lead to a higher risk of dropping out of follow-up.(Table 1).

**Table 1:** Clinical and sociodemographic characteristics of patients with drug-resistant TB in Loreto, 2015–2023.

Variables		n	%	Unsuccessful	%
	< 18 years	34	8.2	13	3.1
Age*	18 - 30 years	93	22.3	65	15.6
	31 - 45 years	113	27.1	55	13.2
	46 - 60 years	109	26.1	50	12.0
	> 60 years	68	16.3	23	5.5
Sex	Female	154	36.9	68	16.3
	Male	263	63.1	138	33.1
TB-HIV coinfection	No	352	84.4	164	39.3
	Yes	43	10.3	29	7.0
	Not evaluated	22	5.3	13	3.1
TB-DM comorbidity	No	319	76.5	160	38.4
	Yes	76	18.2	32	7.7
	Not evaluated	22	5.3	14	3.4
TB treatment category at enrollment	New case	227	54.4	97	23.3
	Relapse	30	7.2	16	3.8
	Treatment after loss to follow-up	67	16.1	46	11.0
	Failure	93	22.3	47	11.3
Delay in treatment initiation	< 14 days	360	86.3	178	42.7
	> 14 days	50	12	21	5.0
	Missing data	7	1.7	7	1.7
TB location	Pulmonary	405	97.1	197	47.2
	Extrapulmonary	12	2.9	9	2.2
Bacillary load	Negative	135	32.4	55	13.2
	Paucibacilar	2	0.5	1	0.2
	+	121	29	60	14.4
	++	75	18	41	9.8
	+++	60	14.4	34	8.2
	Not performed	24	5.8	15	3.6
Alcoholism	No	396	95	189	45.3
	Yes	21	5	17	4.1
Drug addiction	No	395	94.7	187	44.8
	Yes	22	5.3	19	4.6
Resistance	Others	26	6.2	16	3.8
	Hr-TB	74	17.7	18	4.3
	Rr-TB	98	23.5	39	9.4
	PZAr-TB	1	0.2	1	0.2
	Fluoroquinolones resistant-TB	2	0.5	1	0.2
	MDR-TB	216	51.8	131	31.4
District	Periphery	53	12.7	25	6.0
	Maynas city	364	87.3	181	43.4
Treatment scheme	Standardized	48	11.5	35	8.4
	Empiric	276	66.2	127	30.5
	Individualized	93	22.3	44	10.6
	**Total**	**417**		**206**	

* *The mean age of the cohort was 41.7 years (IQR: Q3 (55)-Q1 (27) =28 years)*

**Fig 2:**
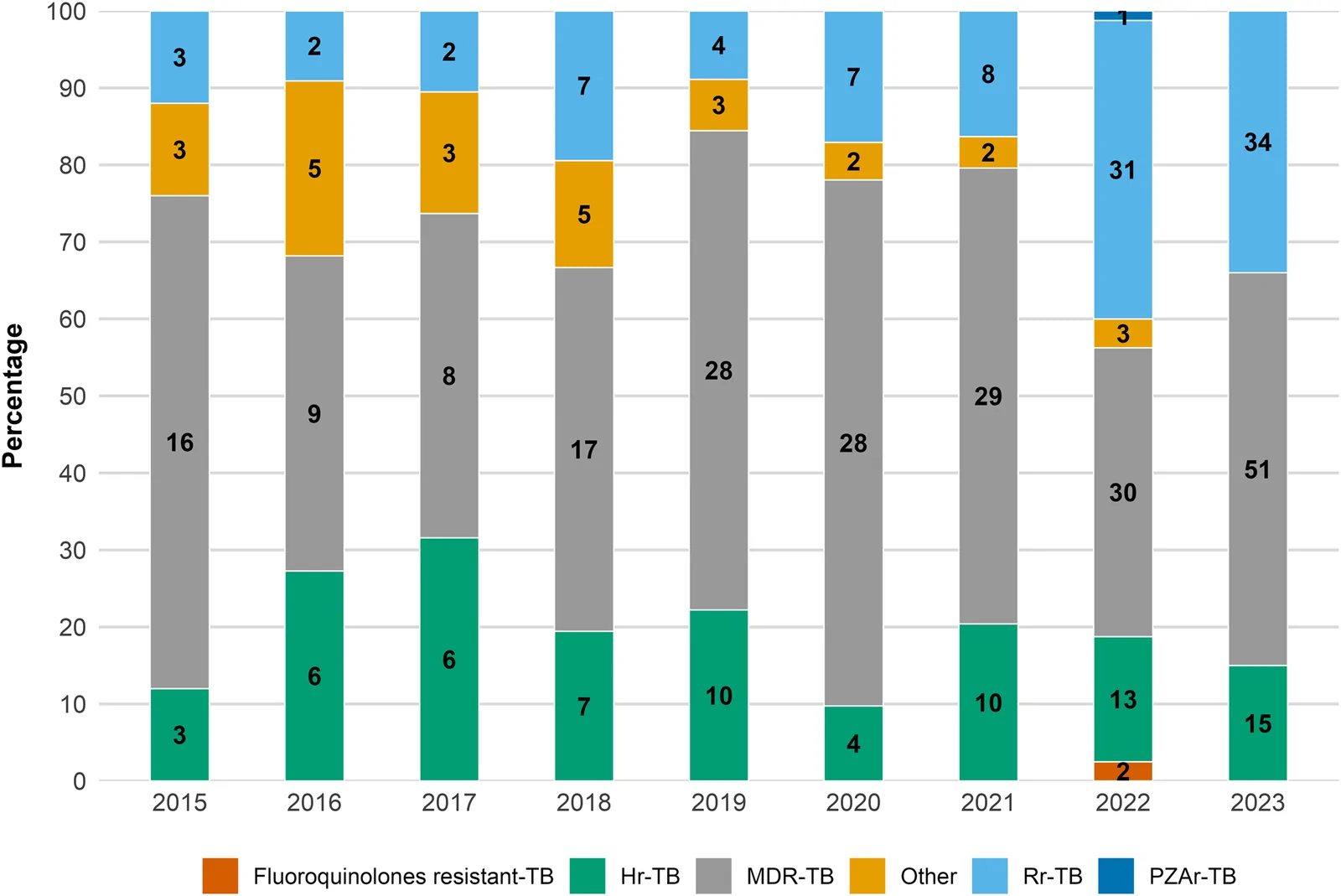
Annual distribution of drug-resistance patterns among patients in Loreto, 2015–2023.

Over the study period, the annual distribution of resistance patterns among DR-TB cases changed substantially (Fig 2). The total number of notified DR-TB cases increased from 25 in 2015–100 in 2023, with a marked rise from 2018 onwards. MDR-TB was the most frequent resistance profile overall (51.8%) and its absolute number of cases increased steadily after 2018, reaching a peak of 51 cases in 2023, cases could be influencing the decrease in treatment success rates during the same study period, as this type of resistance is more complex and prevalent in our region. Rr-TB (n = 98) and Hr-TB (n = 74) were also common and showed low counts in the early years, followed by an increase from 2021 onwards. In contrast, fluoroquinolone-resistant TB, Pyrazidamine (PZA)r-TB and other resistance patterns remained rare, with only isolated cases throughout the study period.

### Treatment Outcomes

**Fig 3:**
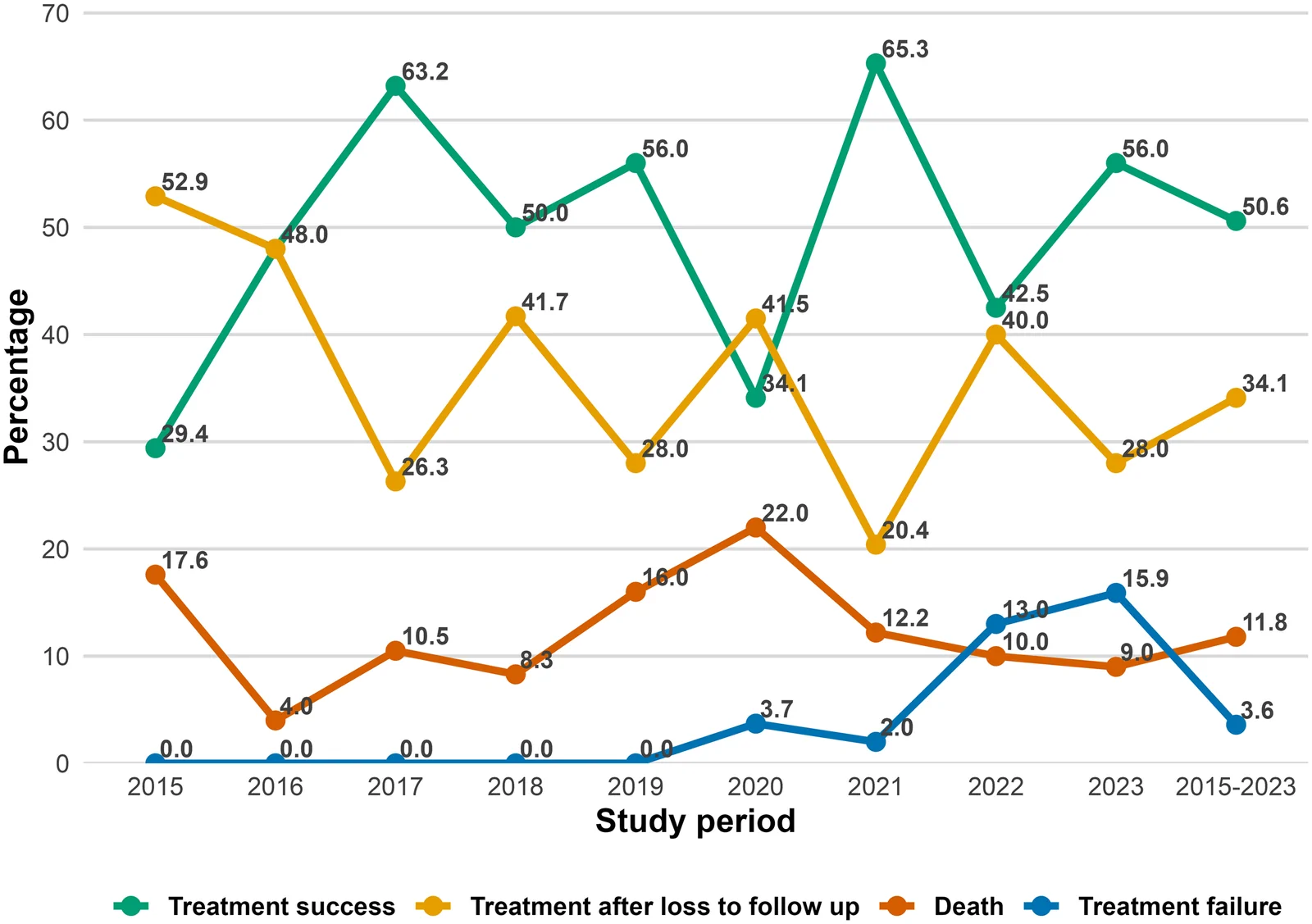
Annual distribution of treatment outcomes among drug-resistant tuberculosis patients in Loreto, 2015–2023.

Of the 417 patients analyzed, 211 (50.6%) achieved treatment success, while 206 (49.4%) experienced unsuccessful outcomes. Within the latter group, loss to follow-up was the most frequent cause (34%), followed by mortality (11.8%) and treatment failure (3.6%). The trend in treatment outcomes in Loreto between 2015 and 2023 (Fig 3) revealed significant program instability. The pre-molecular baseline (2015: 29% success rate, 53% loss to follow-up) improved following the introduction of GeneXpert MTB/RIF (2018: 50% success rate, 42% loss to follow-up), although molecular diagnosis alone was insufficient to ensure adherence. The COVID-19 pandemic was accompanied by a further decline (2020: 34% success rate, 42% loss to follow-up), followed by a temporary recovery in 2021 (65% success rate, 20% loss to follow-up) that proved unsustainable and deteriorated again in 2022 (43% success rate, 40% loss to follow-up). The 2023 cohort (56% success, 28% loss) suggests a post-pandemic stabilization characterized by a programmatic bifurcation: therapeutic efficacy is preserved among retained patients, but structurally high dropout rates persist.

Bivariate logistic regression showed significant associations between unsuccessful treatment outcomes and the following variables: age 18–30 years (OR: 3.8; CI: 1.6–8.5; p = 0.002); TB-HIV coinfection (OR: 2.4; CI: 1.2–4.6; p = 0.012); history of treatment after default (“treatment after loss to follow-up”) (OR: 2.9; CI: 1.6–5.2; p < 0.001); high bacillary load (+++) (OR: 1.9; CI: 1.0–3.5; p = 0.041); alcoholism (OR: 4.7; CI: 1.5–14.1; p = 0.006); drug addiction (OR: 7.0; CI: 2.1–24.2; p = 0.002). In addition, Hr-TB (OR: 0.2; CI: 0.1–0.5; p = 0.001); empiric regimen (OR: 0.3; CI: 0.2–0.6; p = 0.001) and individualized regimen (OR: 0.3; CI: 0.2–0.7; p = 0.004), they were shown to be protective factors, individualized regimens that reduced the probability of failure by 70% (adjusted OR: 0.3)

According to the multivariable analysis, significant associations were found with a history of alcoholism (OR: 3.8; CI: 1.1–13.2; p = 0.037), empiric regimen (OR: 0.5; CI: 0.2-1; p = 0.039) and individualized regimen (OR: 0.4; CI: 0.2–0.8; p = 0.018) (Table 2).

**Table 2:** Factors Associated with Unsuccessful Outcomes in Patients with DR-TB in Loreto, 2015–2023.

Variables			Bivariate analysis		Multivariable analysis	
	Successn (%)	Unsuccessful n (%)	Unadjusted OR (95% CI)	p-value	aOR (95% IC)	p-value
**Age**						
**<** 18 years	21 (5.0)	13 (3.1)	1		1	
18 - 30 years	28 (6.7)	65 (15.6)	**3.8 (1.6-8.5)**	**0.002**	2.3 (0.9-5.9)	0.080
31 - 45 years	58 (13.9)	55 (13.2)	1.5 (0.7-3.4)	0.286	1.0 (0.4-2.5)	0.977
46 - 60 years	59 (14.2)	50 (12.0)	1.4 (0.6-3.0)	0.435	0.8 (0.3-2.0)	0.641
> 60 years	45 (10.8)	23 (5.5)	0.8 (0.4-1.9)	0.660	0.7 (0.3-1.9)	0.504
**Sex**						
Female	86 (20.6)	68 (16.3)	1			
Male	125 (30.0)	138 (33.1)	1.4 (0.9-2.1)	0.102	1.2 (0.7-1.9)	0.479
**TB-HIV coinfection**						
No	188 (45.1)	164 (39.3)	1		1	
Yes	14 (3.4)	29 (7.0)	**2.4 (1.2-4.6)**	**0.012**	2.9 (0.9-9.3)	0.076
Not evaluated	9 (2.2)	13 (3.1)	1.7 (0.7-4.0)	0.259	2.4 (0.6-10.2)	0.222
**TB-DM comorbidity**						
No	159 (38.1)	160 (38.4)	1			
Yes	44 (10.6)	32 (7.7)	0.7 (0.4-1.2)	0.208		
Not evaluated	8 (1.9)	14 (3.4)	1.7 (0.7-4.3)	0.226		
**TB treatment category at enrollment**						
New case	130 (31.2)	97 (23.3)	1		1	
Relapse	14 (3.4)	16 (3.8)	1.5 (0.7-3.3)	0.274	1.2 (0.5-2.9)	0.654
Treatment after loss to follow-up	21 (5.0)	46 (11.0)	**2.9 (1.6-5.2)**	**<0.001**	1.8 (0.9-3.6)	0.121
Failure	46 (11.0)	47 (11.3)	1.4 (0.8-2.2)	0.203	1.3 (0.8-2.4)	0.323
Delay in treatment initiation*						
< 14 days	182 (43.7)	178 (42.7)	1			
> 14 days	29 (7.0)	21 (5.0)	0.7 (0.4-1.3)	0.325		
Missing data	0	7 (1.7)				
**TB location**						
Pulmonary	208 (49.9)	197 (47.2)	1		1	
Extrapulmonary	3 (0.7)	9 (2.2)	3.2 (0.8-11.9)	0.087	4.0 (0.9-17.7)	0.068
**Bacillary load**						
Negative	80 (19.2)	55 (13.2)	1		1	
Paucibacilar	1 (0.2)	1 (0.2)	1.5 (0.1-23.8)	0.793		
+	61 (14.6)	60 (14.4)	1.4 (0.9-2.3)	0.156	1.3 (0.7-2.4)	0.424
++	34 (8.2)	41 (9.8)	1.8 (1.0-3.1)	0.053	1.7 (0.9-3.5)	0.131
+++	26 (6.2)	34 (8.2)	**1.9 (1.0-3.5)**	**0.041**	1.8 (0.8-3.8)	0.147
Not performed	9 (2.2)	15 (3.6)	2.4 (1.0-6.0)	0.052	1.8 (0.6-5.5)	0.311
**Alcoholism**						
No	207 (49.6)	189 (45.3)	1		1	
Yes	4 (1.0)	17 (4.1)	**4.7 (1.5-14.1)**	**0.006**	**3.8 (1.1-13.2)**	**0.037**
**Drug addiction**						
No	208 (49.9)	187 (44.8)	1		1	
Yes	3 (0.7)	19 (4.6)	**7.0 (2.1-24.2)**	**0.002**	3.9 (1.0-15.5)	0.056
Resistance**						
Others	10 (2.4)	16 (3.8)	1		1	
Hr-TB	56 (13.4)	18 (4.3)	**0.2 (0.1-0.5)**	**0.001**	0.3 (0.1-1.0)	0.050
r-TB	59 (14.2)	39 (9.4)	0.4 (0.2-1.0)	0.051	0.7 (0.2-2.0)	0.491
PZAr-TB	0	1 (0.2)				
Fluoroquinolones resistant-TB	1 (0.2)	1 (0.2)	0.6 (0.03-11.2)	0.749	1.5 (0.1-30.2)	0.796
MDR-TB	85 (20.4)	131 (31.4)	1.0 (0.4-2.2)	0.930	1.3 (0.5-3.5)	0.570
**District**						
Periphery	28 (6.7)	25 (6.0)	1			
Maynas city	183 (43.9)	181 (43.4)	1.1 (0.6-2.0)	0.728		
**Treatment scheme**						
Standardized	13 (3.1)	35 (8.4)	1		1	
Empiric	149 (35.7)	127 (30.5)	**0.3 (0.2-0.6)**	**0.001**	**0.5 (0.2-1.0)**	**0.039**
Individualized	49 (11.8)	44 (10.6)	**0.3 (0.2-0.7)**	**0.004**	**0.4 (0.2-0.8)**	**0.018**
**Interaction TB-HIV coinfection/Bacillary load**						
yes/+					0.4 (0.1-3.1)	0.412
yes/++					0.2 (0.01-1.7)	0.134
yes/+++					0.1 (0.01-1.0)	0.051
*N°Obs: 410	*Interaction:*	*Prob>chi2:*			*N°Obs: 411	
**N°Obs: 416		*0.3457*			*LR chi2 [28]: 90.91*	
					*Prob>chi2: 0.0000*	
					*Pseudo R2: 0.1596*	

Given that loss to follow-up accounted for one-third of all unsuccessful outcomes, we conducted an exploratory sub-analysis restricted to patients with treatment success or loss to follow-up (S1 Table). In this model, age 18–30 years (aOR 4.0; 95% CI 1.3-12.3) and TB-HIV coinfection (aOR 3.6; 95% CI 1.0–12.6) was associated with higher odds of loss to follow-up. Compared with other resistance patterns, Hr-TB (aOR 0.2; 95% CI 0.04–0.6) and Rr-TB (aOR 0.3; 95% CI 0.1-0.9) showed a protector factors of loss to follow-up. Individualized treatment regimens were associated with substantially lower odds of loss to follow-up than standardized regimens (aOR 0.3; 95% CI 0.1–0.8). In bivariate analyses, patients with 18–30 years, a history of previous loss to follow-up, and those reporting alcohol and drugs use had higher odds of loss to follow-up, but these associations were attenuated and lost statistical significance after adjustment. Other covariates, including sex, HIV and DM comorbidity, treatment history, delay in treatment history, TB location, smear grade and district, were not significantly associated with loss to follow-up, with wide CIs indicating limited precision.

## Discussion

This study found that approximately half (49.4%) of DR-TB patients treated in the Loreto region between 2015 and 2023 experienced unsuccessful treatment outcomes (including loss to follow-up, treatment failure, or mortality), a figure far below the target set at the national level [13]. These findings are broadly consistent with systematic reviews of MDR and extensively drug-resistant (XDR)-TB from predominantly low-resource settings, which have reported unfavorable outcomes in more than two-thirds of XDR-TB patients and treatment success rates of approximately 25% [17,18]. Among the main factors associated with unfavorable outcomes identified in our multivariable analysis is alcoholism, highlighting the urgent need to strengthen TB control strategies in amazonian settings characterized by clinical and social factors that hinder treatment adherence and therapeutic success.

Alcohol use emerged as key factor associated with unsuccessful therapeutic outcomes, with up to a fourfold higher risk compared to patients without these behaviors. These findings are consistent with previous studies, which reported that illicit substance use is associated with a twofold increase in the risk of unfavorable outcomes, such as loss to follow-up, among patients with DR-TB [19,20]. Alcohol consumption exerts a multifactorial negative impact, being linked to a higher probability of loss to follow-up, more contagious clinical forms such as cavitary TB, and impaired immune response against *M. tuberculosis*, all of which compromise the effectiveness of the pharmacological regimen [21]. In addition, these behaviors impair cognitive function, making it more difficult to sustain adherence to the prescribed pharmacological regimen [22–24]. At the national level, current regulations stipulate that patients who screen positive for alcohol or illicit substance dependence should be referred to the mental health unit of their corresponding health facility, and if specialized care is required, to a higher-level facility for management. However, despite the implementation of this management protocol since 2013, treatment failure rates in this group remain high. In our cohort, information on alcohol and drug use was obtained from routine program records, and standardized screening tools for substance use were not consistently documented across facilities; under-reporting and misclassification of these behaviors are therefore likely, which may have led us to underestimate their true impact on treatment outcomes, therefore, alcohol consumption is a critical factor whose impact could be much greater, and this underscores the urgent need for targeted interventionsThis indicates that barriers persist in the country’s care policies, particularly in relation to comprehensive care and better management of state-provided economic resources, which in turn reduces service quality and coverage, disproportionately affecting the most vulnerable sectors of the Peruvian population. This has been compounded by limited capacity, lack of awareness, and insufficient political will in the areas of mental health and addictions, within a context of scarce economic, human, and technical resources and an overall lack of coverage to address the problem and meet these urgent needs [25]. At present, Peru does not implement TB-specific conditional cash transfer programs or transportation vouchers linked to DR-TB treatment in Loreto, and linkages with existing social protection schemes remain limited; representing a barrier to treatment adherence, particularly regarding transportation costs to health centers that patients face within the city. Studies demonstrate the financial impact of MDR-TB on patients with financial and employment difficulties, revealing that most must quit their jobs, sell belongings, or move to another residence to afford treatment ([26,27]), like the transportation costs for sending samples from the periphery to the regional capital for tuberculosis diagnosis also represent an indirect barrier to adherence to treatment;expanding such mechanisms could help reduce the economic barriers to adherence faced by patients with DR-TB in this setting. Evidence from other settings shows that integrating TB care with addiction and mental health services have improved adherence and treatment completion among people with substance use disorders in other settings. Adapting and evaluating such approaches in Amazonian DR-TB programs could be a promising strategy to reduce loss to follow-up in this high-risk group [28,29]. This underscores the need for a multidisciplinary approach that comprehensively addresses factors such as precarious living conditions, immune system compromise, and poor treatment adherence, in order to improve outcomes in this vulnerable population.

Another significant factor identified in our study was individualized schemes, which proved to be a protective factor, demonstrating that individualized regimens lasting between 9 and 18 months have better treatment outcomes, and accordance with WHO guidelines, treatment regimens for DR-TB patients vary based on factors such as the drug resistance profile, prior exposure to anti-TB medications, patient history, age, TB location, and other criteria [30]. This effectiveness may be due to the application of personalized plans tailored to specific patient characteristics, creating an effective combination for each individual, unlike standard treatment, which uses a fixed set of medications. A meta-analysis in eight studies on MDR-TB patients reported a therapeutic success rate of 67.2% with individualized treatments, alongside rates of treatment failure, dropout, and mortality rates of 8.4%, 13%, and 8.4%, respectively. Conversely, five studies evaluating standardized regimens showed a pooled success rate of 56.9%. These findings highlight that individualized treatment improves the selection of effective drugs, which can result in a high therapeutic success rate for patients by preventing the emergence of new resistant strains [18]. However, this factor should be treated with caution, as there could be selection bias. Individualized treatment would not be applied to patients who experienced early mortality or discontinued treatment, and this would erroneously distort the observed results.

In the bivariate analysis, several factors associated with unfavorable treatment outcomes were found, such as age between 18–30 years, TB-HIV coinfection, a history of treatment abandonment or recovery and high bacillary load (+++). However, when we re-specified the multivariable model to include the treatment regimen variable and a set of prespecified interaction terms, only alcohol use remained independently associated with unsuccessful outcomes. The attenuation of the other associations observed in the bivariable analyses likely reflects confounding, correlations between predictors and limited statistical power in some strata. As previously described, studies support the independent association of alcoholism with adverse TB outcomes, indirectly affecting treatment results through behavioral, biological, and immunological mechanisms ([31]). In this context, it is plausible that some of the effect initially observed for age and HIV in the bivariate analysis was partially absorbed by behavioral variables such as alcoholism in the adjusted model. Furthermore, methodological studies in TB have indicated that clinically relevant variables can lose significance in multivariate models due to collinearity, mediation, or the available sample size, especially in cohorts with multiple interrelated determinants [31].

Young adults (18–30 years) constitute a particularly vulnerable population to unfavorable therapeutic outcomes in TB management [32]. A possible explanation lies in the demands associated with active working life, as they often engage in informal or unstable employment, which limits their availability to consistently attend health services. A labor market analysis conducted in Loreto reported that most young people work in informal jobs without contracts, regulated working hours, or access to social benefits; one in four works independently without professional or technical training, around 30% are employed in microenterprises, and nearly 27% act as unpaid family workers, mainly in rural areas [33]. This situation may be further aggravated by the need to prioritize economic subsistence over treatment continuity, especially in contexts of poverty or limited institutional support. In addition, behavioral and social factors may play a role, as this group tends to have greater mobility and participates in social activities that may involve psychoactive substance use and frequent contact with other people in crowded spaces, factors that not only heighten the risk of acquiring the disease but also of failing to adhere adequately to treatment once diagnosed [34–37]. This interpretation is especially relevant considering that alcohol consumption continues to represent a significant public health problem in young populations. According to a study of 1,882 schoolchildren from public and private institutions, alcohol was the most consumed substance, with an annual prevalence of 11.4%. It is particularly concerning that less than half of the schoolchildren perceived their alcohol consumption as frequent. These findings suggest that substance use patterns may begin early and become entrenched in later stages of life, indirectly contributing to adverse outcomes in chronic diseases such as TB, especially in Amazonian contexts marked by social barriers and access to health care [38]. Targeted peer-support initiatives and community health-worker–led adherence support tailored to young adults could help address these age-specific barriers and reduce loss to follow-up in this group.

The literature mentions, and it is also known, that patients with TB-HIV are at risk of unsuccessful outcomes [23,24,39]. This condition involves a decrease in the patient’s immune system, generating complications and side effects due to the medication burden from both diseases; in addition to the stigma and/or discrimination that the patient may face, which in turn may lead to non-adherence to treatment [40–43].

Although more than 50% of the registered cases were new to anti-TB treatment, the bivariate analysis found a significant association with patients who had discontinued previous anti-TB treatment, as reported in other studies [39,44]. The deterioration of these patients’ health after treatment makes them more vulnerable to developing resistance to anti-TB drugs, which complicates treatment and increases the likelihood of adverse outcomes, including death. Furthermore, some authors point out that many patients abandon treatment when they feel better due to the disappearance of symptoms, creating a false sense of cure that leads them to assume they are already cured and that further treatment is unnecessary [45,46].

It is worth emphasizing that the high proportion of DR-TB among new cases suggests that primary transmission of resistant strains, rather than resistance acquired during previous treatment, may be playing an increasingly important role in Loreto [47]. Similar concerns have been raised in other Amazonian settings, where molecular and genomic studies have identified clustered MDR-TB strains compatible with recent community transmission of resistance [48,49]. Delays in access to drug-susceptibility testing and to adequate regimens may further facilitate ongoing transmission before DR-TB is diagnosed [27]. Nevertheless, regardless of whether the patient had a prior history of TB treatment or was a new case [39], this phenomenon reflects the circulation of resistant strains within the community, underscoring the urgent need to decentralize and strengthen drug susceptibility testing in Loreto [37].

A high bacterial load indicates a large number of bacteria present in the body, which can hinder complete eradication of the infection, as a higher bacterial load may require more prolonged and intensive treatment. Furthermore, a high bacterial load is associated with a greater likelihood of treatment failure and relapses due to the persistence of bacteria in the body despite extensive treatment. A high bacterial load also increases the emergence of resistance to antituberculosis drugs [30]. The interaction between TB-HIV coinfection and high bacterial load (+++) showed a borderline p-value in the final multivariate model (p = 0.051), but it has strong biological plausibility. In HIV-positive patients, a positive smear (+++) reflects advanced immunodeficiency with uncontrolled mycobacterial replication, a phenotype consistently associated with faster clinical deterioration and higher mortality in HIV-associated TB. This apparent synergistic effect between immunosuppression and high infectious load underscores the need for early intensified intervention, rapid initiation of antiretroviral therapy, and closer integration of HIV and TB services in Amazonian settings, where delays in diagnosis can exacerbate this dual pathology. We interpret this interaction with caution, given its borderline significance and the small sample size; however, its consistency with the established immunopathology of HIV-associated TB supports its programmatic relevance for identifying high-risk patients [50].

Likewise, HIV patients were almost four times more likely to drop out of treatment (adjusted OR: 3.7) in the sub-analysis of the 34.1% of patients with discharge status lost to follow-up (the main determinant of unfavorable outcomes), suggesting the need for better integration of HIV/TB services.

The lack of association in diabetic patients in our study may be related to the limited availability of HIV and DM screening for all patients, due to logistical issues and shortages [51], resulting in underreporting of these data in the system. Fasting plasma glucose has high but imperfect sensitivity and specificity for diabetes, so some individuals with true DM may have been classified as non-diabetic. HIV and DM status were documented for 395 of 417 patients (94.7%), while 22 (5.3%) were recorded as “not evaluated.” These variables were obtained from routine program records rather than from systematic study screening, so incomplete testing and recording are likely; consequently, some patients with HIV infection or DM may have been misclassified as negative or “not evaluated,” which could have attenuated the observed associations.

The strengths of this study include the cohort size and the use of population-based data from a region with limited geographic access to drug susceptibility testing, which provides representative evidence for dispersed and hard-to-reach populations. Nevertheless, the study was subject to limitations such as the use of routinely collected program data, including several self-reported variables such as alcohol or drug use, which may be underreported or misclassified. For relatively infrequent exposures, such as alcohol or drug use, this was reflected in wide confidence intervals around some estimates, indicating limited precision and warranting cautious interpretation of these associations. In addition, DR-TB diagnosis, notification and treatment are concentrated in Maynas province, where most of the regional population and diagnostic capacity are located; patients who were never diagnosed, never notified, or who remain in remote riverine communities and do not reach referral centers in Iquitos are likely under-represented, leading to potential case-ascertainment bias. District of residence was included only as a crude contextual variable, grouped as Maynas versus peripheral districts. This classification may mask important within-district heterogeneity, including differences between urban, peri-urban, rural, and riverine populations, as well as actual travel time or distance to health facilities. In addition, the study period spanned nine years, during which diagnostic tools, treatment regimen and programmatic policies for DR-TB evolved; such temporal changes may have influenced both exposure distributions and outcomes, and were not fully accounted for in the analysis, so some degree of temporal bias is possible. Several potentially important predictors, such as mental health conditions, social support, distance and travel time to clinics, or clinic-level characteristics, were not systematically recorded and therefore could not be explored. Consequently, residual confounding by unmeasured factors, including nutritional status, household overcrowding, distance to and accessibility of health facilities, and details of treatment regimen composition, cannot be excluded. Furthermore, district residence was included as a crude contextual variable (Maynas city versus peripheral districts) and likely masks substantial within-district heterogeneity. These area-level measures should therefore be interpreted as proxies of contextual disadvantage rather than individual-level determinants, and inferences may be affected by ecological fallacy. No formal sensitivity analyses were conducted, which may limit the assessment of the robustness of some associations. The study was conducted in Loreto, a rural Amazonian region with high poverty, geographic isolation and limited health-system capacity, so the findings are most applicable to similar resource-limited settings and may not generalize to urban areas with wider access to DR-TB diagnostics and individualized care, although the patterns observed in young adults and in people who use alcohol or drugs may extend to other high-burden regions. Finally, the retrospective cohort study design, which evaluates predictors and end-of-treatment outcomes at a single time point, does not allow us to establish causality, and all associations should be interpreted as non-causal.

## Conclusions

Given that approximately half of patients with DR-TB experience unfavorable treatment outcomes, the current centralized care model is insufficient to address the complex geographic, social, and cultural realities of Loreto. These findings highlight the urgent need to implement a comprehensive TB strategy adapted to the Amazonian context, based on decentralizing services through mobile units and community-based detection and follow-up teams, strengthening access to rapid drug susceptibility testing and providing adherence support in riverine and hard-to-reach areas, and systematically integrating mental health and the management of alcohol and drug use disorders into DR-TB care. Furthermore, strengthening social support, increasing the participation of community health workers, and promoting peer support could significantly contribute to improving treatment adherence and outcomes. Finally, future prospective and genomic studies will be essential to evaluate the effectiveness of these contextualized interventions and to better understand the impact of primary transmission and other social and clinical determinants on DR-TB outcomes in Amazonian settings.

## Supporting information

S1 Table Factors associated with loss to follow-up versus treatment success among patients with drug-resistant tuberculosis in Loreto, 2015–2023. (XLSX)

S1 Data De-identified minimal data set used in the analyses. (XLSX)
