# Correction: Factors associated with unsuccessful treatment outcomes among drug-resistant tuberculosis patients in Loreto, Peru, 2015–2023: A retrospective cohort study

**DOI:** 10.1371/journal.pgph.0007559

**Published:** 2026-10-28

**Authors:** Evelyn Genthell Cordova-Pisco, Tery Vasquez-Hassinger, Estela Huamán-Angeles, Karine Zevallos, George Obregon

The ORCID iDs are missing for the third and fourth author. Please see the authors’ respective ORCID iDs here:

- Author Estela Huamán-Angeles’s ORCID iD is: 0000-0002-7462-1083 (https://orcid.org/0000-0002-7462-1083).
- Author Karine Zevallos’s ORCID iD is: 0000-0001-5205-4970 (https://orcid.org/0000-0001-5205-4970).

## References

[1] Cordova-Pisco EG, Vasquez-Hassinger T, Huamán-Angeles E, Zevallos K, Obregon G. Factors associated with unsuccessful treatment outcomes among drug-resistant tuberculosis patients in Loreto, Peru, 2015–2023: A retrospective cohort study. PLOS Glob Public Health 2026;6(9):e0007229. doi: 10.1371/journal.pgph.0007229 PMC13557377 42715181

